# Lamin B1 levels modulate differentiation into neurons during embryonic corticogenesis

**DOI:** 10.1038/s41598-017-05078-6

**Published:** 2017-07-07

**Authors:** Sameehan Mahajani, Caterina Giacomini, Federica Marinaro, Davide De Pietri Tonelli, Andrea Contestabile, Laura Gasparini

**Affiliations:** 1 Dept. of Neuroscience and Brain Technologies, Istituto Italiano di Tecnologia, Genova, Italy; 2 Universitaetsmedizin Goettingen, Waldweg 33, Goettingen, 37073 Germany; 3 Division of Cancer Studies, King’s College London, New Hunt’s House, Guy’s Campus, London, SE1 1UL UK; 4 Abbvie Deutschland GmbH & Co, Knollstr, Ludwigshafen 67061 Germany

## Abstract

Lamin B1, a key component of the nuclear lamina, plays an important role in brain development. Ablation of endogenous Lamin B1 (*Lmnb1*) in the mouse strongly impairs embryonic brain development and corticogenesis. However, the mechanisms underlying these neurodevelopmental effects are unknown. Here, we report that Lamin B1 levels modulate the differentiation of murine neural stem cells (NSCs) into neurons and astroglial-like cells. *In vitro*, endogenous Lmnb1 depletion favors NSC differentiation into glial fibrillar acidic protein (GFAP)-immunoreactive cells over neurons, while overexpression of human Lamin B1 (*LMNB1*) increases the proportion of neurons. In *Lmnb1*-null embryos, neurogenesis is reduced, while *in vivo Lmnb1* silencing in mouse embryonic brain by in utero electroporation of a specific *Lmnb1* sh-RNA results in aberrant cortical positioning of neurons and increased expression of the astrocytic marker GFAP in the cortex of 7-day old pups. Together, these results indicate that finely tuned levels of Lamin B1 are required for NSC differentiation into neurons, proper expression of the astrocytic marker GFAP and corticogenesis.

## Introduction

Lamin B1 is a major component of the nuclear lamina, and together with other lamins (e.g., lamin A/C), plays a role in the structure and function of the nucleus^1^. Although ubiquitous, Lamin B1 is highly expressed in the rodent brain^2–5^. Defects in the gene encoding this protein have been associated with diseases mainly affecting the central nervous system^6–9^. The murine (*Lmnb1*) and human Lamin B1 (*LMNB1*) genes apparently act as genetic modifiers, affecting the risk of neural tube defects in mice^10^ and humans^8^. Further, in humans, a genomic duplication of *LMNB1* is thought to cause adult-onset autosomal dominant leukodystrophy (ADLD)^6, 7^, the first identified laminopathy that affects the central nervous system^11^. In a family with an ADLD variant, a genomic deletion upstream of the *LMNB1* gene has been also shown to induce changes in genetic regulatory mechanisms, leading to up regulation of LMNB1 protein and disease manifestation^9^.

The pathological phenotypes associated with Lamin B1 abnormalities suggest that this protein is essential for proper brain development and function in rodents and humans. Indeed, Lmnb1 protein levels vary during neurogenesis in rodents^12^, consistent with a potentially stage-specific or dose-dependent role. In one patient with an ADLD variant, the expression of LMNB1 is specifically enhanced in degenerating cerebral areas^9^, supporting the view that changes in LMNB1 levels have deleterious consequences for the brain. Consistently, overexpression of Lamin B1 in the mouse brain is associated with abnormal neuronal activity, microglial reaction, astrogliosis and myelin abnormalities^13, 14^. We have recently demonstrated that Lamin B1 is required for proper morphological differentiation of dendrites in primary mouse cortical neurons *in vitro* ^15^. Further, in constitutive *Lmnb1* knockout mice, Lmnb1 deficiency results in perinatal lethality, reduced brain size, abnormal layering and apoptosis of cortical neurons^16–18^, while forebrain-specific Lmnb1 knockout results in reduced cortex, decreased density of cortical neurons and lack of upper cortical layers^17^. These findings indicate that levels of Lamin B1 are critical for mouse cortical development. However, the cellular and molecular mechanisms by which lamin B1 levels regulate corticogenesis and neural stem cell (NSC) differentiation are still largely unclear.

Here, we started to address this fundamental question by investigating how Lamin B1 expression levels regulate neuronal differentiation during embryonic corticogenesis. Using primary neural stem cells (NSCs) and gain-of-function (overexpressing *LMNB1*) and loss-of-function (*Lmnb1* deficiency) approaches, we find that Lamin B1 levels regulate the balance of differentiation into neurons versus astrocytic-like cells *in vitro*. Specifically, the loss of Lmnb1 favors NSC differentiation into glial fibrillar astrocytic protein (GFAP)-positive (GFAP^+^) cells over neurons, without affecting oligodendrocyte generation. These effects also occur in the embryonic brain: in Lmnb1-null (*Lmnb1* ^*Δ*/*Δ*^) mice^16^, embryonic neurogenesis is reduced. Further, Lmnb1 knockdown *in vivo* by in utero electroporation of a specific Lmnb1 sh-RNA plasmid results in increased expression of the astrocytic markers GFAP in the area of silenced cells. Overall, this work demonstrates that finely tuned levels of Lamin B1 are required for NSCs to differentiate in proper numbers of neurons and express cell-type specific genes during corticogenesis.

## Results

### Lamin B1 levels balance neuronal versus astrocytic differentiation in cultured primary mouse NSCs

**Figure 1:**
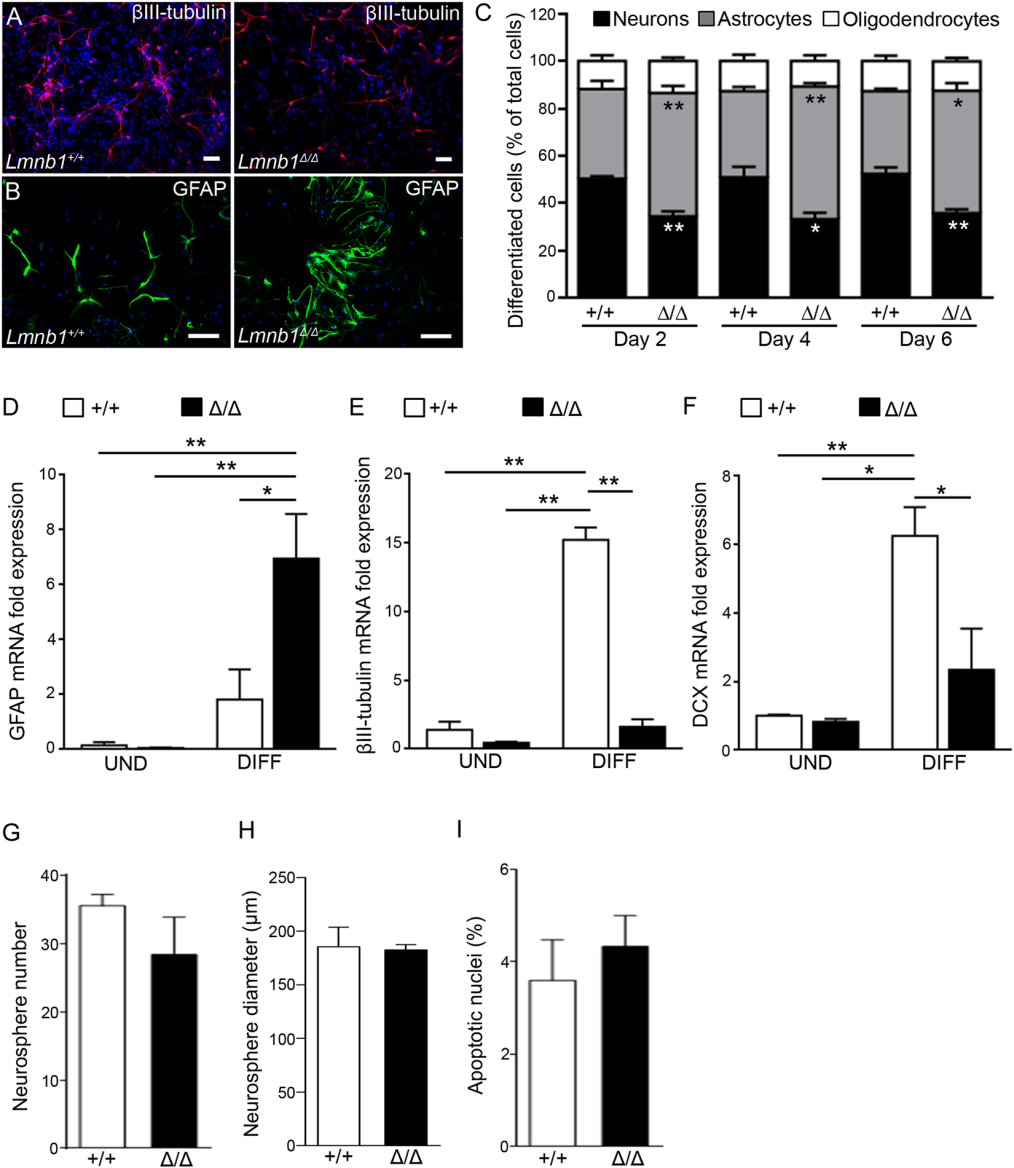
*Lmnb1* deficiency alters NSC differentiation into neurons and astrocytes. NSCs were cultured from *Lmnb1* ^+/+^ and *Lmnb1* ^*Δ*/*Δ*^ embryos and differentiated for 2, 4 or 6 days. (**A**,**B**) Fluorescence images of immunoreactivity for βIII-tubulin (**A**; red) and GFAP (**B**; green) in NSCs differentiated for 4 days. Nuclei are counterstained with DAPI (blue). Scale bars: 50 µm. (**C**) Quantitative analysis of differentiated cells. Data represent the percentage of neurons, astrocytes, and oligodendrocytes out of the total number of cells. *p < 0.05, **p < 0.01 vs Lmnb1^+/+^ at the respective differentiation time, Student’s t-test. Approximately 1000 cells for each staining condition were quantified in 3 independent experiments. (**D**–**F**) Quantitative analysis of mRNA expression of cellular markers GFAP (**D**), βIII-tubulin (**E**) and DCX (**F**) in NSCs from *Lmnb1* ^+/+^ and *Lmnb1* ^*Δ*/*Δ*^ in an undifferentiated state or after differentiation for 4 days. *p < 0.05, **p < 0.01, two-way ANOVA followed by Bonferroni post hoc test. (**G**,**H**) Quantitative analysis of NSC self-renewal. Number (**G**) and diameter (**H**) of neurospheres were measured 72 h after seeding single cell suspensions of equal numbers of cells (200 cells/embryo). (**I**) Quantitative analysis of pyknotic nuclei in *Lmnb1* ^+/+^ and *Lmnb1* ^*Δ*/*Δ*^ NSCs. A total of 300 cells were counted. In all graphs, bars represent the average ± SEM from 3 independent experiments.

**Figure 2:**
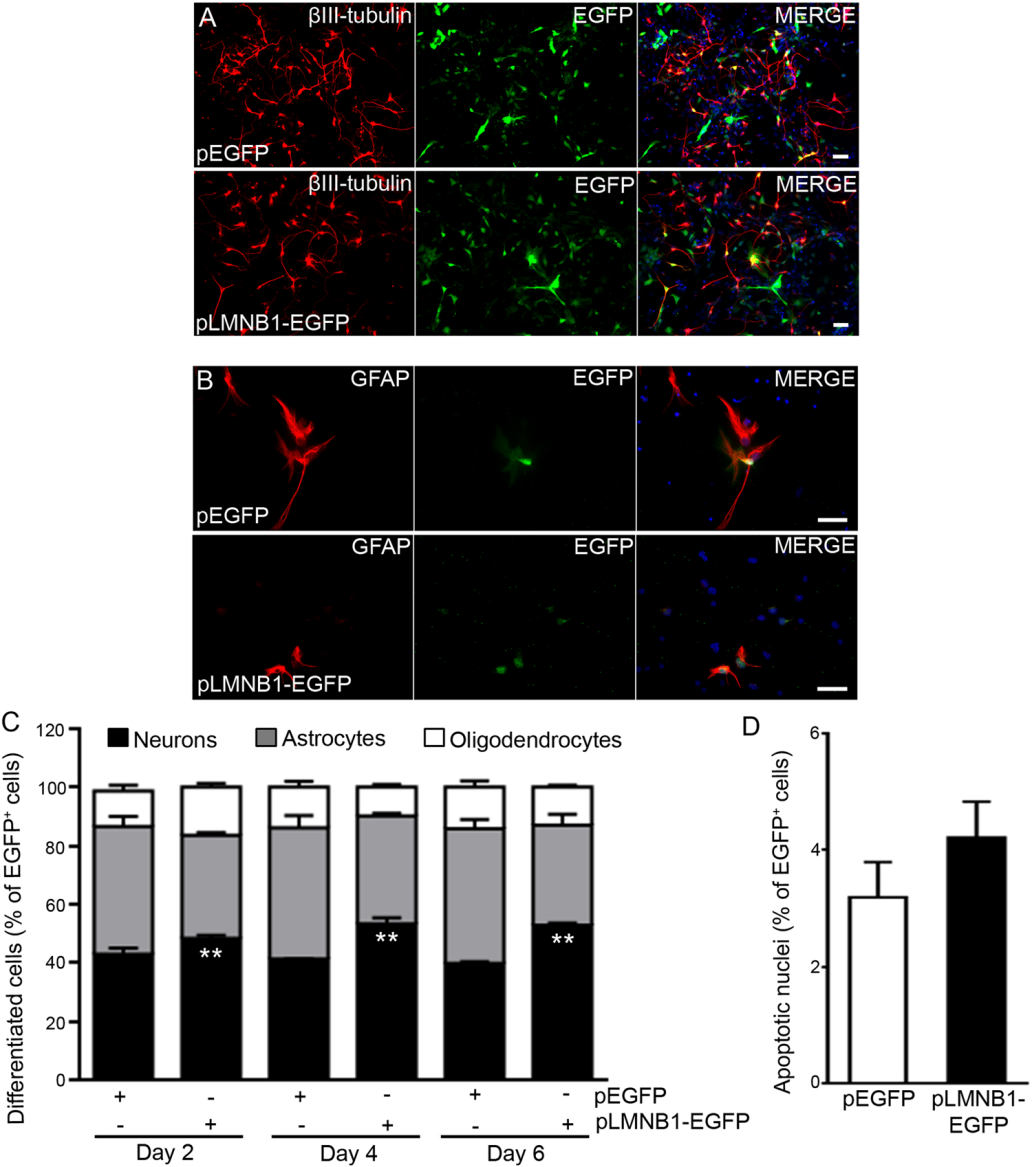
LMNB1 overexpression increases the proportion of NSC differentiation into neurons. NSCs from C57BL6/J embryos were transfected with pEGFP or pLMNB1-EGFP and differentiated for 2, 4 or 6 days. (**A**–**D**) Fluorescence images of immunoreactivity for βIII-tubulin (**A**,**B**; red), GFAP (**C**,**D**; red) and EGFP (**A**–**D**; green) in NSCs differentiated for 4 days. Nuclei are counterstained with DAPI (blue). Scale bars: 50 µm. (**E**) Quantitative analysis of differentiated cells. Data represent the percentage of neurons, astrocytes, and oligodendrocytes co-expressing EGFP out of the total number of EGFP^+^ cells. *p < 0.05, **p < 0.01 vs pEGFP transfected cells at the respective differentiation time, Student’s t-test. Approximately 400 EGFP^+^ cells were quantified for each staining condition in 3 independent experiments. (**F**) Quantitative analysis of NSC pyknotic nuclei. 150 cells per condition were counted. In all graphs, bars represent the average ± SEM from 3 independent experiments.

To investigate whether Lmnb1 affects NSC differentiation, we differentiated primary E11.5 NSCs lacking endogenous *Lmnb1* (Fig. 1) or overexpressing *LMNB1* (Fig. 2) using defined culture conditions for 2, 4 or 6 days. We then analyzed the differentiated cells by immunostaining for cell-specific markers: βIII-tubulin for neurons (Figs 1A and 2A), GFAP for astrocytes (Figs 1B and 2B) and PDGFRα for oligodendrocyte precursors (Supplementary Fig. S1A,B).

In *Lmnb1* ^*Δ*/*Δ*^ cultures, the percentage of βIII-tubulin-positive (βIII-tubulin^+^) cells was significantly reduced (Fig. 1A,C), while the number of GFAP^+^ cells was increased compared to *Lmnb1* ^+/+^ cultures (Fig. 1B,C). The percentage of PDGFRα-positive (PDGFRα^+^) cells was similar in *Lmnb1* ^*Δ*/*Δ*^ and *Lmnb1* ^+/+^ cultures differentiated for 2, 4 and 6 days (Fig. 1C). In undifferentiated *Lmnb1* ^+/+^ and *Lmnb1* ^*Δ*/*Δ*^ NSCs, levels of GFAP and βIII-tubulin mRNA were similar (Fig. 1D,E). However, differentiated *Lmnb1* ^*Δ*/*Δ*^ cells consistently had higher GFAP mRNA levels and lower βIII-tubulin mRNA levels than *Lmnb1* ^+/+^, as judged by qRT-PCR (Fig. 1D,E). Further, the expression of doublecortin (DCX), which marks post-mitotic newborn neurons in the first 3 weeks of age^19^, was not statistically different in undifferentiated *Lmnb1* ^*Δ*/*Δ*^ cells compared to *Lmnb1* ^+/+^ (Fig. 1F). When NSCs were differentiated for 4 days, the expression of DCX increased in *Lmnb1* ^+/+^ cells, but remained at levels similar to undifferentiated NSCs in *Lmnb1* ^*Δ*/*Δ*^ cells (Fig. 1F), indicating reduced neuronal differentiation in the absence of *Lmnb1*.

We next investigated whether this change in the proportions of derivative cell types depends on effects of Lamin B1 levels on the proliferation and survival of progenitors. To examine whether the absence of Lmnb1 affects proliferation, we assessed NSCs’ ability to generate secondary neurospheres over 72 h using a standard clonogenic assay^20, 21^. We found that both *Lmnb1* ^+/+^ and *Lmnb1* ^*Δ*/*Δ*^ NSCs produced equal numbers of secondary neurospheres (Fig. 1G) of comparable size (Fig. 1H), indicating that NSCs’ self-renewal properties are conserved in the absence of Lmnb1. We then examined cell death in undifferentiated *Lmnb1* ^*Δ*/*Δ*^ NSCs: the number of pyknotic nuclei was similar in *Lmnb1* ^+/+^ and *Lmnb1* ^*Δ*/*Δ*^ (Fig. 1I), indicating that absence of Lamin B1 protein do not affect the survival of E11.5 neuronal progenitors *in vitro*.

To evaluate the effects of increased LMNB1 levels, we transfected undifferentiated C57BL6/J NSCs with *LMNB1* and the *EGFP* reporter or *EGFP* alone, and after differentiation, analyzed the number of cells double-positive for EGFP and βIII-tubulin (Fig. 2A,B), GFAP (Fig. 2C,D) or PDGFRα (Supplementary Fig. S1C,D). LMNB1 overexpression significantly enhanced the percentage of EGFP-positive (EGFP^+^)/βIII-tubulin^+^ cells at all differentiation time points (Fig. 2E) with a slight but not significant reduction in the proportions of EGFP^+^/GFAP^+^ cells and EGFP^+^/PDGFRα^+^ cells compared to differentiated cells expressing EGFP alone. The number of pyknotic nuclei was similar in NSCs transfected with both LMNB1 and/or EGFP (Fig. 2D), indicating that up regulation of Lamin B1 protein do not affect the survival of E11.5 neuronal progenitors *in vitro*.

Together, these results indicate that *in vitro*, the neuronal differentiation varies in direct proportion to Lamin B1 levels and suggest that *Lmnb1* depletion favors NSC differentiation into GFAP^+^ cells over neurons, without affecting survival or self-renewal properties of undifferentiated NSCs.

### Lmnb1 deficiency reduces neurogenesis in the developing embryonic brain

**Figure 3:**
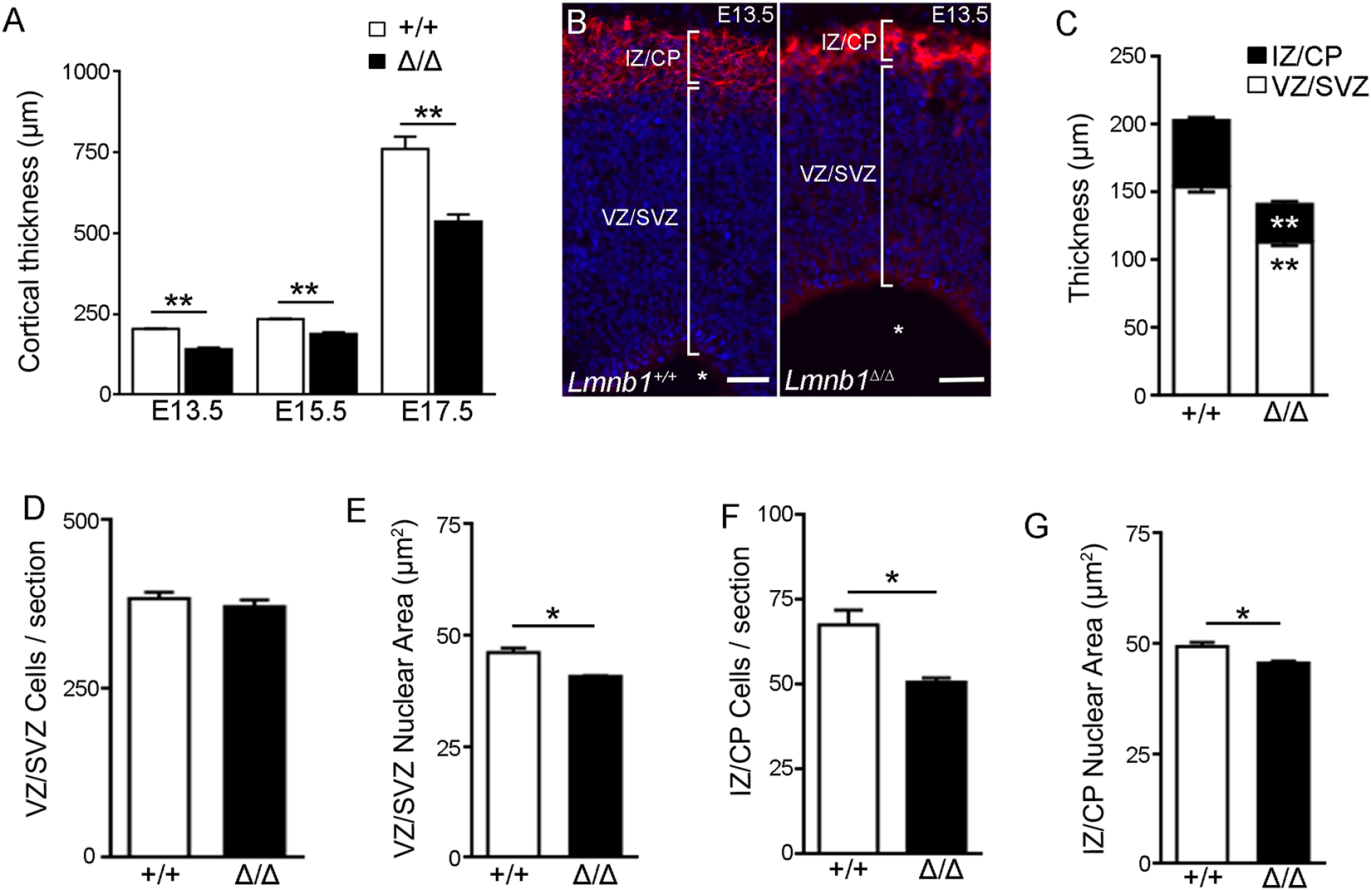
Lmnb1 deficiency significantly impairs corticogenesis and neurogenesis during embryonic development. (**A**) Quantitative analysis of cortical thickness in brain sections from *Lmnb1* ^+/+^ and *Lmnb1* ^*Δ*/*Δ*^ embryos at E13.5, E15.5 and E17.5. (**B**) Fluorescence images of cortical coronal sections of E13.5 *Lmnb1* ^+/+^ and *Lmnb1* ^*Δ*/*Δ*^ brains immunostained for βIII-tubulin (red) and counterstained with Hoechst 33342 (blue). Scale bars: 20 µm. (**C**) Quantitative analysis of the thickness of the IZ/CP and VZ/SVZ in E13.5 brains. **p < 0.01 vs respective brain area of Lmnb1^+/+^. (**D**) Cell counts in the VZ/SVZ in E13.5 brains. (**E**) Nuclear area of VZ/SVZ cells in E13.5 brains. (**F**) Cell counts in the IZ/CP in E13.5 brains. (**G**) Nuclear area of IZ/CP cells in E13.5 brains. In all graphs, bars represent the average ± SEM from 3 independent experiments. *p < 0.05, **p < 0.01, Student’s t-test.

Previous work demonstrated that the overall cortical thickness is reduced in *Lmnb1* ^*Δ*/*Δ*^ embryos at E15.5–17.5^17^. We confirmed this finding and further report that cortical thickness is already decreased at E13.5 (Fig. 3A). In the mouse telencephalon, neuronal progenitors of the ventricular zone (VZ)/subventricular (SVZ) start differentiating into neurons around E10–11, populating the intermediate zone (IZ)/cortical plate (CP)^22^. To detail how Lmnb1 affects neuronal progenitors and neurogenesis *in vivo*, we thus analyzed the VZ/SVZ and IZ/CP in *Lmnb1* ^*Δ*/*Δ*^ and *Lmnb1* ^+/+^ embryos at E13.5. Brain sections were immunostained for the neuronal marker βIII-tubulin to visualize the intermediate zone (IZ) and CP, and nuclear DNA was counterstained with Hoechst 33342. The VZ/SVZ was identified as the region close to the ventricle and devoid of βIII-tubulin. The thickness of both the IZ/CP and VZ/SVZ was reduced in the E13.5 *Lmnb1* ^*Δ*/*Δ*^ brain (Fig. 3B,C). However, the average percentage reduction of the IZ/CP was greater (−41%) than that of the VZ/SVZ (−22%), suggesting that Lmnb1 absence impairs newborn neurons generation with relative retention of neural progenitors.

To test this hypothesis, we counted the cells present in the IZ/CP and VZ/SVZ of E13.5 embryonic cortices of *Lmnb1* ^*Δ*/*Δ*^ and *Lmnb1* ^+/+^ mice. At E13.5, *Lmnb1* ^*Δ*/*Δ*^ and *Lmnb1* ^+/+^ had a similar number of VZ/SVZ cells (Fig. 3D). However, in *Lmnb1* ^*Δ*/*Δ*^ brain, VZ/SVZ cells appeared more densely packed due to reduced nuclear area compared to *Lmnb1* ^+/+^ (Fig. 3E). In the IZ/CP, E13.5 *Lmnb1* ^*Δ*/*Δ*^ embryos had significantly fewer βIII-tubulin^+^ neurons (Fig. 3F) than *Lmnb1* ^+/+^. IZ/CP *Lmnb1* ^*Δ*/*Δ*^ neurons also had reduced nuclear area (Fig. 3G), but such reduction is smaller (−8%) than that of VZ/SVZ (−14%) cells.

These results indicate that during early brain development, the absence of Lamin B1 affects cell density, nuclear area and impairs neuronal differentiation without affecting the overall number of VZ/SVZ progenitors.

### Knockdown of Lmnb1 *in vivo* induces aberrant positioning of Lmnb1-silenced neurons and increased GFAP expression in a subset of cells

**Figure 4:**
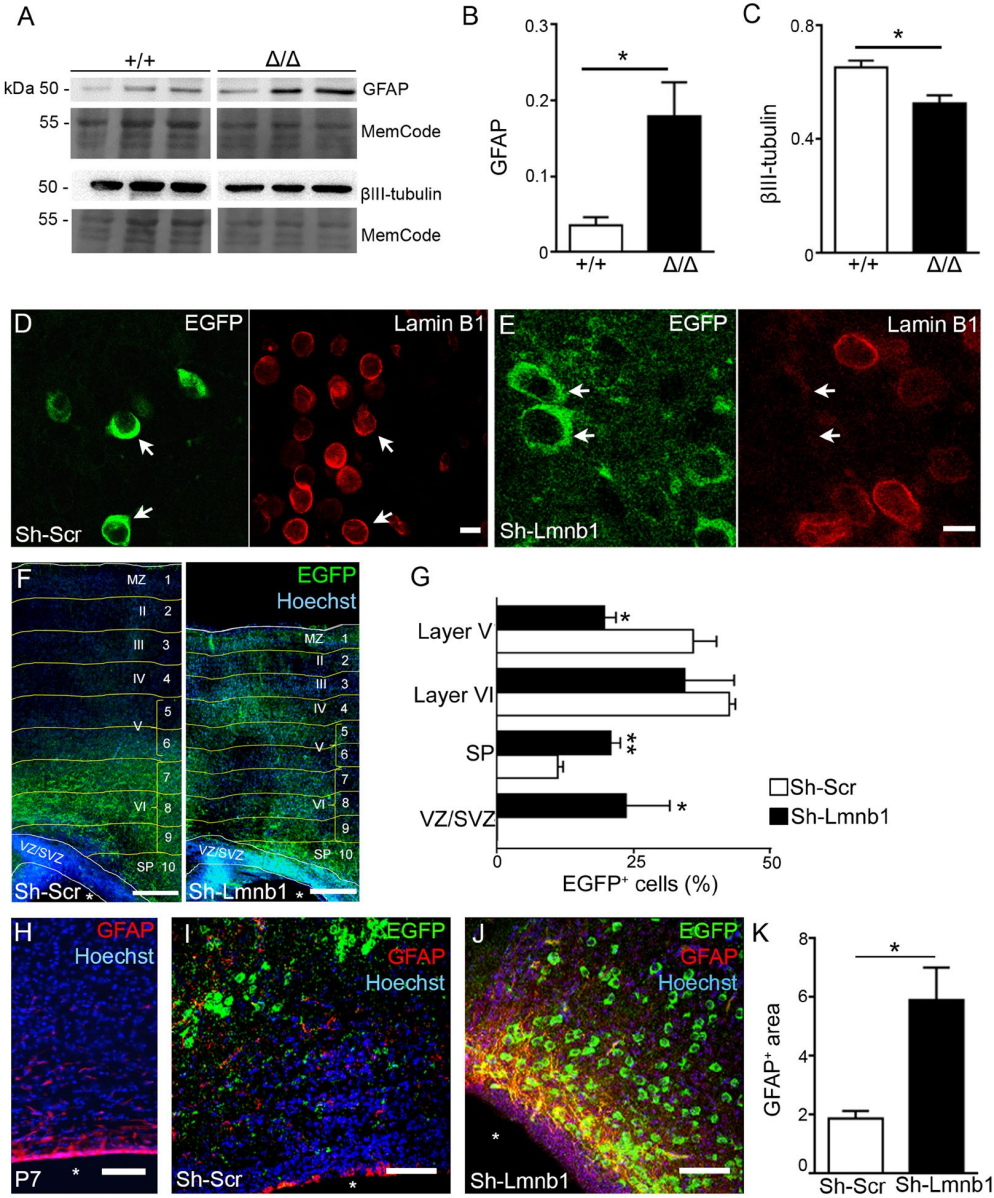
Knockdown of Lmnb1 increases GFAP protein expression *in vivo*. GFAP protein expression was analyzed in brain homogenates of *Lmnb1* ^*Δ*/*Δ*^ and *Lmnb1* ^+/+^ embryos or in brain sections of P7 C57BL6/J pups after Lmnb1 silencing. (**A**) Western blot analysis of GFAP, βIII-tubulin, Lmnb1 and actin. (**B**,**C**) Quantitative analysis of GFAP (**A**,**B**) and βIII-tubulin (**A**,**C**) in E17.5 *Lmnb1* ^*Δ*/*Δ*^ and *Lmnb1* ^+/+^ brain lysates. Data are normalized on MemCode. Graph bars represent the average ± SEM. n = 3/genotype, *p < 0.05; **p < 0.01, Student’s t-test. Cropped blots presented in Fig. 4A and full-length blots are presented in Supplementary Fig. S5. (**D**,**E**) Maximal projections of confocal z-stack images of immunoreactivity for Lmnb1 (red) and EGFP (green) in P7 brains after electroporation with sh-Scr (**D**) and sh-Lmnb1 (**E**) Arrows indicate corresponding electroporated cells. Scale bars: 5 µm. (**F**) Fluorescence confocal images of immunoreactivity for EGFP (green) in P7 brains electroporated with sh-Scr and sh-Lmnb1 plasmids. Scale bars: 50 µm. White lines delimit the VZ/SVZ and the cortical superficial boundary. Yellow lines delineate bins (Hevner *et al*.^40^), which are identified by Arabic numbers. Roman numerals indicate cortical layers I-VI. MZ, marginal zone; SP, sub-plate. Nuclei were counterstained by Hoechst 33342 dye (blue); white asterisks identify the ventricle. (**G**) Quantitative analysis of EGFP^+^ cells in P7 brains as a function of their position in the cortex after electroporation with sh-Scr and sh-Lmnb1. Graph bars represent the average percentage of EGFP^+^ cells/layer ± SEM from 3 independent experiments. *p < 0.05, **p < 0.01 vs respective layer in sh-Scr, Student’s t-test. (**H**) Fluorescence confocal images of immunoreactivity for GFAP (red) in naïve P7 C57BL6/J brain. (**I**,**J**) Maximal projections of confocal z-stack images of immunoreactivity for GFAP (red) and EGFP (green) in P7 C57BL6/J brains electroporated with sh-Scr (**I**) or sh-Lmnb1 (**J**). In (**H**–**J**), nuclei are counterstained with Hoechst 33342 (blue). White asterisks identify the ventricle. Scale bars: 100 µm. (**K**) Quantitative analysis of area immunoreactive for GFAP in electroporated brains. GFAP^+^ area is expressed as percentage of section area. Graph bars represent the average ± SEM, n = 3; *p < 0.05, Student’s t-test.

At the late embryonic and early postnatal stages (E16–18), cortical NSCs start differentiating into astrocytes^23, 24^. To examine whether Lmnb1 deficiency affects astrocytic differentiation *in vivo*, we analyzed GFAP and βIII-tubulin protein expression in the brain of *Lmnb1* ^*Δ*/*Δ*^ and *Lmnb1* ^+/+^ E17.5 embryos (Fig. 4). Consistent with the results in NSC cultures (Figs 1 and 2), there is a significant reduction of βIII-tubulin protein levels in *Lmnb1* ^*Δ*/*Δ*^ brain (Fig. 4A,C), while GFAP levels were significantly higher in *Lmnb1* ^*Δ*/*Δ*^ than *Lmnb1* ^+/+^ brain (Fig. 4A,B). This suggests that Lmnb1 deficiency causes an untimely increase of protein expression of such astrocytic marker.

In the mouse cortex, astrogliogenesis starts during late embryonic development, but the majority of astrocytes are produced during the early postnatal period from proliferation of resident astrocytes of embryonic origin^25^. Accordingly, we did not detect any GFAP immunoreactivity in the brain from both *Lmnb1* ^+/+^ and *Lmnb1* ^*Δ*/*Δ*^ E13.5 embryos (Supplementary Fig. 3A,B). At E19.5, some GFAP^+^ cells were observed in the SVZ of both *Lmnb1* ^+/+^ and *Lmnb1* ^*Δ*/*Δ*^ (Supplementary Fig. 3C,D). Consistent with biochemical analysis in whole brain lysate, GFAP immunoreactivity was more intense in *Lmnb1* ^*Δ*/*Δ*^ than *Lmnb1* ^+/+^ brain. In *Lmnb1* ^+/+^ brain, the morphology of GFAP+ cells differs between E19.5 and P7 (Supplementary Fig. 3E), suggesting different degree of maturation.

*Lmnb1* ^*Δ*/*Δ*^ mice die at birth^16^. Thus, to investigate whether the depletion of Lmnb1 in embryonic neural progenitors also affects postnatal GFAP expression *in vivo*, we knocked down Lmnb1 expression in VZ/SVZ progenitors of C57BL6/J embryos by in utero electroporation of mouse specific *Lmnb1* sh-RNA (sh-Lmnb1) or scramble sh-RNA (sh-Scr) plasmids (Supplementary Fig. 2A,B) in E14.5 embryos and analyzed the pups at postnatal day 7 (P7). Lmnb1 expression, EGFP^+^ cells positioning and GFAP expression were examined in electroporated brains. Both the sh-Scr and sh-Lmnb1 constructs had the same electroporation efficiency and achieved equal numbers of EGFP+ cells (Supplementary Fig. S2C,D). sh-Lmnb1, but not sh-Scr, specifically knocks down endogenous Lmnb1 (Fig. 4D,E), without affecting the total number of cells nor inducing cell death (Supplementary Fig. S2E,F). However, consistent with previous birth-dating findings showing that absence of Lmnb1 affects neuronal migration in the *Lmnb1* ^*Δ*/*Δ*^ embryonic brain^17^, we found an aberrant positioning of Lmnb1-silenced neurons. Neurons born at E14 typically localize in layer IV–V^26^; in mice electroporated with sh-Scr control plasmid, EGFP^+^ cells correctly localized in layer V (Fig. 4F,G). In contrast, in mice where Lmnb1 is silenced by sh-Lmnb1, over 40% of EGFP^+^ cells localized across the VZ/SVZ and the sub-plate (SP), with only a reduced percentage of EGFP^+^ cells in layer V (Fig. 4F,G). In terms of GFAP expression, brains electroporated with sh-Scr (Fig. 4I) were similar to C57BL6/J non-electroporated (Fig. 4H): GFAP immunoreactivity was very low and limited to a few cells in the SVZ and scattered cells and short processes through the cortex with no overlap with EGFP^+^ cells (Fig. 4I). Lmnb1 silencing by sh-Lmnb1 electroporation resulted in the disappearance of GFAP signal at the SVZ rim and induced a strong increase in GFAP immunoreactivity in thick cell processes and areas adjacent to EGFP^+^ cells (Fig. 4J). Consistently, the area of GFAP immunoreactivity significantly increased by 3-fold in brains with Lmnb1 silencing (Fig. 4K). The different subcellular distribution of EGFP (mostly somatic) and GFAP (cell processes) prevented a precise quantification of the number of cells immunoreactive for both markers. Nevertheless, some of the processes were immunoreactive for both GFAP and EGFP, indicating that at least a subset of EGFP^+^ cells were also expressing GFAP.

Overall, these results suggest that the knock down of Lmnb1 induces untimely expression of the astrocytic marker GFAP during embryonic and postnatal cortical development *in vivo* and aberrant positioning of Lmnb1-silenced cortical neurons.

## Discussion

Lamin B1 plays a key role in embryonic development and organogenesis in the mouse^16–18^ and is essential for brain corticogenesis^17, 18^. The effects of Lmnb1 are dependent on its cerebral levels: conditional knockout of Lmnb1 in the brain is sufficient to recapitulate the developmental phenotype of constitutive knockout mice^17^. Here we now demonstrate that Lmnb1 influences the expression of cell-type specific genes and the differentiation of neural progenitors. At the cellular level, *in vitro*, the rate of differentiation into neurons increases in direct proportion to Lamin B1 levels, while *Lmnb1* depletion favors NSC differentiation into GFAP^+^ cells, without affecting progenitor survival or self-renewal properties of undifferentiated NSCs. These effects also occur in the embryonic brain *in vivo*: in *Lmnb1* ^*Δ*/*Δ*^ embryos, neurogenesis is reduced at E13.5 and the expression of GFAP is increased from E17.5. Further, embryonic Lmnb1 knockdown *in vivo* by in utero electroporation of a specific Lmnb1 sh-RNA plasmid increases the postnatal expression of the astrocyte marker GFAP in some silenced cells and adjacent area.

The effects of Lmnb1 during development vary across organs, developmental stage and cell type. Indeed, while Lmnb1 deficiency strongly affects the development of brain, heart and lung^16–18^, absence of both B-lamins does not influence the skin and hair development^27^. These developmental effects of Lmnb1 possibly results from cell-type specific actions on cell differentiation, proliferation or senescence^16, 27–30^. In peripheral cells, absence of Lmnb1 prevents the differentiation of mouse embryonic fibroblasts (MEFs) into adipocytes^16^, but has no effect on the differentiation of keratinocytes^27^. It has also been recently reported that Lamin B1 is required for proper differentiation of adult, murine olfactory sensory neurons and expression of mature neuron-specific genes^31^. Our results now show that during early embryonal corticogenesis, despite retaining the ability to differentiate into different neural cell types, NSCs with abnormal levels of Lmnb1 generate altered proportions of neurons and induce increased expression of the astrocytic marker GFAP.

In agreement with findings in primary glial postnatal cultures^5^, LMNB1 overexpression does not significantly affect the proportion of NSC-derived GFAP^+^ cells. Conversely, Lmnb1-deficiency significantly increases the proportion of NSC-derived GFAP^+^ cells *in vitro*. Further, Lmnb1 knock down *in vivo* induces GFAP expression in cells of the sub-plate. At least a subset of EGFP^+^ Lmnb1-silenced cells also express GFAP, as indicated by the detection of cellular processes immunoreactive for both GFAP and EGFP. However, despite being close to Lmnb1-silenced cells, most processes present only GFAP immunoreactivity. This observation is consistent with data obtained in NSCs *in vitro*, where only a subset of cells differentially expresses neuronal or glial markers. Moreover, these findings suggest that *in vivo*, embryonic Lmnb1 knockdown may also act with non-cell autonomous mechanisms turning on GFAP expression, inducing gliosis and/or proliferation of resident astrocytes nearby Lmnb1-silenced cells. Further investigation is warranted to clarify these effects of Lmnb1 silencing *in vivo*.

In the mouse, Lmnb1 deficiency causes microcephaly and reduces cortical thickness throughout embryonic development^17, 18^. The reduced cortical thickness may result from different processes at defined gestational ages. During early development (E13.5), reduced neurogenesis and reduced nuclear size are mainly observed in Lmnb1-null embryonic brain. In the IZ/CP, both cell number and nuclear area are significantly reduced in Lmnb1-null E13.5 brain, resulting in a thickness percentage reduction (−41%) comparable to the percentage reduction of the overall cortex (−30%). In the Lmnb1-null VZ/SVZ, despite reduced nuclear size, the number of cells is preserved, indicating overall NSC survival. This is consistent with preserved proliferation and survival of cultured Lmnb1-null NSCs and with previous findings demonstrating that Lmnb1 is not essential for proliferation and survival of embryonic stem cells^15, 17, 18^. Instead, at E15.5 and beyond, apoptosis and reduced numbers of neurons have been reported by our and other groups^15, 17, 18^, indicating that additional mechanisms undermine corticogenesis in Lmnb1-null embryos at late developmental stage.

Lmnb1-null cortex displays altered lamination, which has been mainly ascribed to defective migration of cortical newborn neurons^17^. Consistently, we find that when Lmnb1 is silenced, cortical migration of differentiating cells is hampered. Further, in Lmnb1-null brain, neurons are present in the VZ/SVZ area, which is usually devoid of mature neurons^15^. Our previous^15^ and current findings suggest that aberrant morphological differentiation of neurons^15^ and premature expression of astrocytic markers may also contribute to the composite phenotype of Lmnb1 deficiency on brain development. The causal and temporal relationship of differentiation and migration phenotypes induced by Lamin B1 deficiency remains unclear. Further investigation is warranted to elucidate the mutual interactions between neuronal differentiation and migration in the context of Lamin B1 deficiency.

In conclusion, in agreement with previous evidence that abnormal levels of Lamin B1 dysregulate expression and splicing of genes involved in neural development^32^ and maturation^31^, our work suggests that levels of Lamin B1 finely tune corticogenesis by modulating neuronal differentiation of mouse neural progenitors and glial gene expression. Together with previous findings from other groups on effects of Lamin B1 on neuronal migration, these results provide a framework for understanding the neurodevelopmental consequence of loss-of-function mutations in the Lamin B1 gene.

## Materials and Methods

### Animals

Transgenic *Lmnb1* hemizygous (*Lmnb1* ^+/*Δ*^) mice^16^ were obtained from the MMRRC Mutant Mouse Regional Resource Center (Univ. of California, Davis, CA, USA) and backcrossed with C57BL6/J mice (Charles River) for 12 generations. Throughout the study, we used *Lmnb1* ^*Δ*/*Δ*^ embryos and *Lmnb1* homozygous (*Lmnb1* ^+/+^) littermates obtained by crossing *Lmnb1* ^+/*Δ*^. In selected experiments (i.e., in utero electroporation and NSC generation for gain of function studies *in vitro*), C57BL6/J wild type mice and CD1 foster mothers (Charles River) were also used. Mice of both sexes were used. Animal health and comfort were veterinary-controlled. The mice were housed in cages with filters in a temperature-controlled room with a 12:12 hour dark/light cycle with *ad libitum* access to water and food. All animal experiments were performed full compliance with the European Community Council directive dated 86/609/EEC, the revised directive 2010/63/EU and were approved by the Italian Ministry of Health and by the IIT Ethical Committee.

The genotypes of newborns were determined using primers specific for the wild type *Lmnb1* allele (forward, 5′-TCCGTGTCGTGTGGTAGGAGG-3′; reverse, 5′-GCAGGAGGGTTGGGAAAGCC-3′) and the mutant allele carrying the gene-trap insertion (forward, 5′-TCCGTGTCGTGTGGTAGGAGG-3′; reverse, 5′-CACTCCAACCTCCGCAAACTC-3′).

### Antibodies and reagents

The following primary antibodies were used: mouse monoclonal antibodies against EGFP (Millipore, cat. MAB3580; 1:500), rabbit polyclonal antibodies against Lamin B1 (Abcam, cat. 16804; 1:200), βIII-tubulin (Sigma, cat. T-2200; 1:500), GFAP (Dako, cat. Z0334; 1:1000), platelet-derived growth factor receptor-α (PDGFR-α; SantaCruz, cat. 338; 1:250). Secondary antibodies conjugated with Alexa fluorophores (Invitrogen) or horseradish-peroxidase (HRP; Bio-Rad) were used. For nuclear counterstaining, 4′,6′-diamidino-2-phenylindole (DAPI; Invitrogen, cat. D3571; 1:1000) or Hoechst 33342 (Sigma, cat. 14533; 1:1000) were used.

Unless otherwise specified, general reagents and chemicals were from Sigma and reagents for cell cultures were from Invitrogen. Primers are listed in Supplementary Table S1.

### Culture and differentiation of neural stem cells (NSCs)

NSCs were obtained from the dorsal forebrain of *Lmnb1* ^+/+^, *Lmnb1* ^*Δ*/*Δ*^ or C57BL6/J embryos at embryonic day 11.5–12.5 (E11.5–12.5) as in ref. 33. The forebrain was dissected in phosphate-buffered saline (PBS) pH 7.4, dissociated using Accutase® (Sigma) at 37 °C for 6 minutes, triturated into single cells and seeded into Neurobasal medium containing B-27 (1:50 v/v), 1% Pen-Strep, Glutamax, 20 ng/ml Epidermal Growth Factor (EGF; Sigma) and 10 ng/ml basic Fibroblast Growth Factor (bFGF; PeproTech). NSCs were grown at 37 °C in a humidified incubator with 5% CO_2_. Free-floating spherical-shaped clumps of cells known as neurospheres formed in 4–5 days. Neurospheres were passaged every 3 days. After 2 passages, to induce differentiation, neurospheres were triturated into single cells using Accutase® and plated on glass coverslips coated with 0.1 mg/ml Poly L-Lysine (PLL; Sigma) for 2–6 days in medium without growth factors. To examine *Lmnb1* ^+/+^ and *Lmnb1* ^*Δ*/*Δ*^ progenitors’ proliferation, we used a standard clonogenic assay^20, 21^. For each embryo, 200 cells were plated as single cell suspension and cultured over 72 h to assess NSC ability of generating secondary neurospheres. The total number of secondary neurospheres obtained from *Lmnb1* ^+/+^ and Lmnb*1* ^*Δ*/*Δ*^ NSCs was counted. The diameter of these neurospheres was measured using ImageJ software on phase contrast images acquired using Olympus SZX16 microscope with Olympus Digital Cell^A imaging software.

### Cultures of mouse primary neurons and N2a cells

Primary cortical neurons were prepared from E17–18 mouse embryos of wild type (WT) mice as previously described^15^. Neurons were plated onto poly-l-lysine (PLL)-coated petri at 30–40 × 10^4^ cells/petri and cultured in Neurobasal containing B-27, Glutamax, penicillin/streptomycin at 37 °C in a 5% CO_2_ humidified atmosphere. Neuronal N2a cells were cultured as described previously^34^ (Supplementary Fig. S2A,B).

### Plasmids and transfection

pCAG-LMNB1-ires-EGFP (pLMNB1-EGFP) or pCAG-ires-EGFP (pEGFP) plasmids have been described elsewhere^34^. Briefly, human LMNB1 cDNA (NM_005573.3) was cloned under the CMV early enhancer/chicken β-actin (CAG) promoter in the bicistronic vector containing the Enhanced Green Fluorescent Protein (EGFP) reporter downstream to the IRES sequence. NSCs derived from C57BL6/J embryos were transfected using the Amaxa P4 transfection kit and Amaxa 4D-Nucleofector X Unit (DS-113 program; Lonza). LMNB1 was expressed only in NSCs transfected with pLMNB1-EGFP, but not with pEGFP (Supplementary Fig. S4). The efficiency of transfection was similar for both plasmids (percentage of EGFP^+^ cells. pLMNB1-EGFP: 42.4 ± 0.6; pEGFP: 45.2 ± 1.0; P = 0.07, Student t-test).

Mouse Lmnb1 shRNA (clone TRCN0000091906 in pLKO.1‐CMVtGFP™ vector) and scramble shRNA (MISSION® pLKO.1‐CMV‐tGFP™ nontarget shRNA Control) plasmids containing the TurboGFP™ (tGFP; indicated as EGFP in Fig. 4 and related text) reporter were obtained from Sigma. The shRNA sequence and tGFP were under the control of U6 and CMV promoters, respectively. The target sequences for Lmnb1 silencing were as follows: *Lmnb1:* 5′- CCGGGCGTCAGATTGAGTATGAGTACTCGAGTACTCATACTCAATCTGACGCTTTTTG-3′ (Clone ID: NM_010721.1–956s1c1); Scramble: 5′- CAGTATTTCTACGAGACCAACTCGAGTTGGTCTCGTAGAAATACTGC-3′. The loop sequence is 5′-CTCGAG-3′.

Primary neurons were transduced with Sh-Lmnb1, Sh-Scr or mock lentiviruses as described elsewhere^15^ (Supplementary Fig. S2B). N2a cells were transfected by lipofection^34^ (Supplementary Fig. S2A).

### In utero electroporation

*Lmnb1* silencing *in vivo* was carried out by in utero electroporation using Lmnb1 shRNA (Sh-Lmnb1) or scramble-shRNA (Sh-Scr) plasmids as described^35^. The pregnant female C57BL6/J mouse (E14) was anesthetized using isofluorane. After injection of DNA (3 µg) containing 1% Fast-Green dye (Sigma) through the uterine wall into the lumen of the telencephalic vesicles, each embryo was subjected to electroporation by five square electric pulses (30 mV for 50 ms, 1 s intervals) delivered through platinum electrodes (Sonidel) using a BTX-ECM 830 electroporator (Harvard Apparatus). After electroporation, the abdominal cavity was sutured and the pregnant mouse was administered diclofenac (0.5 mg/kg) (Voltaren, Novartis) by intraperitoneal injection and kept warm until awake. For Lmnb1 knockdown studies, electroporated pups were born and immediately transferred to a foster CD-1 mother. The pups were sacrificed at postnatal day 7 (P7) and the brains collected for analysis.

### Quantitative Real-Time PCR (qRT-PCR)

Neurospheres were collected before plating on PLL (undifferentiated NSCs) or after differentiation for 1 or 4 days. RNA was extracted using the RNeasy Micro Kit (Qiagen) and quantified with a Nanodrop-1000 Spectrophotometer (NanoDrop Technologies, Inc). qRT-PCR was performed as described in ref. 36. Complementary DNA was analyzed by qRT-PCR (5 ng RNA equivalent/reaction) using 0.2 µM target-specific primers and QuantiFast SYBR Green (Qiagen). Primers were validated by qRT-PCR analysis on serial dilutions of a positive control complementary DNA to assess their specificity and efficiency. Forty qRT-PCR cycles were performed on 7900HT instrument (Applied Biosystems) equipped with the Sequence Detection Systems (SDS) 2.3 software. Data were normalized using the expression of the housekeeping genes Actin and HPRT1 as in ref. 36. C_t_ values were converted into fold-expression values relative to the control using qBase^PLUS^ software (Biogazelle).

### Immunocytochemistry

Immunocytochemistry was performed as previously described with minor modifications^15, 34^. Briefly, cells were fixed with 4% paraformaldehyde (PFA) in PBS, blocked and permeabilized with 12% normal goat serum (Euroclone) in PBS containing 0.25% Triton X-100 for 45 min. Coverslips were then incubated with primary antibodies diluted in blocking solution for 3 h at room temperature or overnight at 4 °C, followed by Alexa dye-conjugated secondary antibodies. Coverslips were mounted using Prolong Gold anti-fade reagent (Invitrogen) containing DAPI. Fluorescence images were collected using an automated Olympus BX51 microscope, equipped with an MBF Optonic CX9000 camera and 40X UPLFLN semi-apo fluorite NA 0.3 objective and MBF Neurolucida V11 software. Confocal imaging was performed using a Leica TCS SP5 AOBS TANDEM inverted confocal microscope equipped with HCX PL APO 40× 1.25 oil and HCX PL APO blue 63× 1.4 NA oil objectives.

The proportion of *Lmnb1* ^*Δ*/*Δ*^ and *Lmnb1* ^+/+^ NSC-derived differentiated cells was quantified as the percentage of cells positive for neuronal (βIII-tubulin), astrocytic (GFAP) or oligodendrocytic (PDGFR-α) markers over the total number of cells identified by nuclear staining with DAPI. When LMNB1 was overexpressed, the percentage of differentiated cells positive for EGFP and one of the cellular markers were calculated over the total number of EGFP^+^ cells to determine the proportion of differentiation.

Nuclear morphology was analyzed by an experienced researcher on blinded samples. According to previous work, pyknotic nuclei displaying chromatin condensation identified cells undergoing apoptosis^15, 37^ or to some extent, necrosis^38^. To determine the nuclear area, *Lmnb1* ^*Δ*/*Δ*^ and *Lmnb1* ^+/+^ NSCs-derived differentiated cells were stained for neuronal, astrocyte and oligodendrocyte markers and nuclei were counterstained with nuclear dye 4,6-diamidino-2-phenylindole (DAPI). For each cell type, nuclei were manually selected on fluorescence images and the nuclear area was quantified using the ImageJ software^39^.

### Immunohistochemistry

Embryonic or neonatal brains were fixed in 4% paraformaldehyde (PFA) overnight, cryopreserved in 30% sucrose in phosphate buffer saline (PBS) overnight at 4 °C, embedded in Tissue Tek OCT compound (Sakura Finetek) and stored at −80 °C. Coronal cryosections (10 μm, 40 μm or 80 μm) were cut on a Leica CM 3050 S cryostat and kept in PBS with 0.02% sodium azide until used. Section at bregma 1.44 mm with interaural plane at 3.94 mm were selected for analyses. Before staining, the sections were incubated in 4% PFA for 20 min at room temperature. Sections were blocked in 5% normal goat serum in PBS containing 0.25% Triton X-100 for 45 min and incubated with primary antibodies in blocking solution overnight at 4 °C, followed by an appropriate Alexa dye-conjugated secondary antibody. Fluorescence and confocal imaging was performed as described above.

Cortical thickness for *Lmnb1* ^*Δ*/*Δ*^ and *Lmnb1* ^+/+^ embryos at different developmental stages was measured on slices collected at the level of the ventricle and stained with Hoechst 33342 dye as the reference. A straight line was drawn from the SVZ to the marginal zone (MZ) and the length was measured using ImageJ^39^.

For the measurement of IZ/CP and VZ/SVZ thickness, sections from E13.5 *Lmnb1* ^*Δ*/*Δ*^ and *Lmnb1* ^+/+^ embryos were immunostained for βIII-tubulin (neuronal marker) to identify the IZ/CP and counterstained with Hoechst 33342 to visualize the entire cortex. The portion of the cortical plate devoid of βIII-tubulin staining, but with Hoechst positive cells, was considered as the VZ/SVZ.

For counting cells in the VZ/SVZ and IZ/CP, a width of 1000 µm was set for both *Lmnb1* ^*Δ*/*Δ*^ and *Lmnb1* ^+/+^ brain sections collected at the same bregma. Cells were counted throughout the VZ/SVZ (Hoechst positive; βIII-tubulin negative) or IZ/CP (positive for Hoechst and βIII-tubulin) and normalized to the respective area determined using ImageJ.

For the quantification of numbers of electroporated cells located in the cortical layers after *Lmnb1* silencing, we divided the cortical plate into 10 equal bins as previously described^40^. According to this method, in P7 normal rodent cortex (bregma 2.91 mm), bins correspond to MZ (bin 1), cortical layers II-IV (bins 2–4), cortical layer V (bins 5–6) and cortical layer VI (bins 7–9). The last bin (bin 10) corresponds to the sub-plate (SP) with additional bins for the IZ (bin 11) and SVZ (bin 12). The number of EGFP^+^ cells was quantified based on their position in the bins. Hoechst counterstaining was used to identify nuclei. Cells with pyknotic nuclei were counted and identified as apoptotic as previously described^15^.

### Western blotting

*Lmnb1* ^+/+^ and *Lmnb1* ^*Δ*/*Δ*^ E18.5 brains were lysed by sonication in 0.5 M NaCl, 50 mM Tris, 1% sodium dodecyl sulfate (SDS) supplemented with protease and phosphatase inhibitors (Sigma). Equal amounts of protein were separated on 10% bis-tris polyacrylamide gels as in ref. 34. Equal loading was verified using MemCode (Thermo Scientific). Densitometry was performed using ImageJ^39^ and protein levels were normalized to MemCode.

### Statistical analysis

Statistical analysis was performed using GraphPad Prism software. For datasets with normal distribution, the Student’s t-test was used for comparison between two groups. ANOVA followed by a Bonferroni post hoc test was used for comparing more than two groups. The differences between groups were considered statistically significant when p < 0.05. Data throughout the text are reported as average values ± SEM, except when otherwise specified.

## Electronic supplementary material

Supplemental Materials

## References

[1] Zastrow MS, Flaherty DB, Benian GM, Wilson KL (2006). Nuclear titin interacts with A- and B-type lamins *in vitro* and *in vivo*. J. Cell Sci..

[2] Broers JL (1997). A- and B-type lamins are differentially expressed in normal human tissues. Histochem. Cell Biol..

[3] Tunnah D, Sewry CA, Vaux D, Schirmer EC, Morris GE (2005). The apparent absence of lamin B1 and emerin in many tissue nuclei is due to epitope masking. J. Mol. Histol..

[4] Worman HJ, Lazaridis I, Georgatos SD (1988). Nuclear lamina heterogeneity in mammalian cells. Differential expression of the major lamins and variations in lamin B phosphorylation. J. Biol. Chem..

[5] Lin ST, Fu YH (2009). miR-23 regulation of lamin B1 is crucial for oligodendrocyte development and myelination. Dis. Model. Mech..

[6] Padiath QS (2006). Lamin B1 duplications cause autosomal dominant leukodystrophy. Nat. Genet..

[7] Brussino A (2009). A novel family with Lamin B1 duplication associated with adult-onset leucoencephalopathy. J. Neurol. Neurosurg. Psychiatry.

[8] Robinson A (2013). Is LMNB1 a susceptibility gene for neural tube defects in humans? *Birth defects research*. Part A, Clinical and molecular teratology.

[9] Giorgio E (2015). A large genomic deletion leads to enhancer adoption by the lamin B1 gene: a second path to autosomal dominant adult-onset demyelinating leukodystrophy (ADLD). Hum Mol Genet.

[10] De Castro SC (2012). Lamin b1 polymorphism influences morphology of the nuclear envelope, cell cycle progression, and risk of neural tube defects in mice. PLoS genetics.

[11] Cortelli P, Terlizzi R, Capellari S, Benarroch E (2012). Nuclear lamins: Functions and clinical implications. Neurology.

[12] Takamori Y (2007). Differential expression of nuclear lamin, the major component of nuclear lamina, during neurogenesis in two germinal regions of adult rat brain. Eur. J. Neurosci..

[13] Heng MY (2013). Lamin B1 mediates cell-autonomous neuropathology in a leukodystrophy mouse model. The Journal of clinical investigation.

[14] Rolyan H (2015). Defects of Lipid Synthesis Are Linked to the Age-Dependent Demyelination Caused by Lamin B1 Overexpression. The Journal of neuroscience: the official journal of the Society for Neuroscience.

[15] Giacomini C, Mahajani S, Ruffilli R, Marotta R, Gasparini L (2016). Lamin B1 protein is required for dendrite development in primary mouse cortical neurons. Molecular biology of the cell.

[16] Vergnes L, Peterfy M, Bergo MO, Young SG, Reue K (2004). Lamin B1 is required for mouse development and nuclear integrity. Proc. Natl. Acad. Sci. USA.

[17] Coffinier C (2011). Deficiencies in lamin B1 and lamin B2 cause neurodevelopmental defects and distinct nuclear shape abnormalities in neurons. Mol. Biol. Cell.

[18] Kim Y (2011). Mouse B-type lamins are required for proper organogenesis but not by embryonic stem cells. Science.

[19] Vreugdenhil E (2007). Doublecortin-like, a microtubule-associated protein expressed in radial glia, is crucial for neuronal precursor division and radial process stability. The European journal of neuroscience.

[20] Molofsky AV, He S, Bydon M, Morrison SJ, Pardal R (2005). Bmi-1 promotes neural stem cell self-renewal and neural development but not mouse growth and survival by repressing the p16Ink4a and p19Arf senescence pathways. Genes Dev.

[21] Kalani MY (2008). Wnt-mediated self-renewal of neural stem/progenitor cells. Proc Natl Acad Sci U S A.

[22] Taverna E, Gotz M, Huttner WB (2014). The cell biology of neurogenesis: toward an understanding of the development and evolution of the neocortex. Annual review of cell and developmental biology.

[23] Kriegstein A, Alvarez-Buylla A (2009). The glial nature of embryonic and adult neural stem cells. Annu Rev Neurosci.

[24] Juliandi B, Abematsu M, Nakashima K (2010). Epigenetic regulation in neural stem cell differentiation. Dev Growth Differ.

[25] Ge WP, Miyawaki A, Gage FH, Jan YN, Jan LY (2012). Local generation of glia is a major astrocyte source in postnatal cortex. Nature.

[26] Takahashi T, Goto T, Miyama S, Nowakowski RS, Caviness VS (1999). Sequence of neuron origin and neocortical laminar fate: relation to cell cycle of origin in the developing murine cerebral wall. The Journal of neuroscience: the official journal of the Society for Neuroscience.

[27] Yang SH (2011). An absence of both lamin B1 and lamin B2 in keratinocytes has no effect on cell proliferation or the development of skin and hair. Human molecular genetics.

[28] Shimi T (2011). The role of nuclear lamin B1 in cell proliferation and senescence. Genes Dev.

[29] Freund A, Laberge RM, Demaria M, Campisi J (2012). Lamin B1 loss is a senescence-associated biomarker. Molecular biology of the cell.

[30] Dreesen O (2013). Lamin B1 fluctuations have differential effects on cellular proliferation and senescence. The Journal of cell biology.

[31] Gigante CM (2017). Lamin B1 is required for mature neuron-specific gene expression during olfactory sensory neuron differentiation. Nat Commun.

[32] Bartoletti-Stella A (2015). Messenger RNA processing is altered in autosomal dominant leukodystrophy. Hum Mol Genet.

[33] Currle, D. S., Hu, J. S., Kolski-Andreaco, A. & Monuki, E. S. Culture of mouse neural stem cell precursors. *Journal of visualized experiments: JoVE* 152, doi:10.3791/152 (2007). 10.3791/152 PMC2532938 18830426

[34] Ferrera D (2014). Lamin B1 overexpression increases nuclear rigidity in autosomal dominant leukodystrophy fibroblasts. FASEB journal: official publication of the Federation of American Societies for Experimental Biology.

[35] De Pietri Tonelli D (2006). Single-cell detection of microRNAs in developing vertebrate embryos after acute administration of a dual-fluorescence reporter/sensor plasmid. BioTechniques.

[36] Ferrera D, Mazzaro N, Canale C, Gasparini L (2014). Resting microglia react to Abeta42 fibrils but do not detect oligomers or oligomer-induced neuronal damage. Neurobiology of aging.

[37] Taylor RC, Cullen SP, Martin SJ (2008). Apoptosis: controlled demolition at the cellular level. Nature reviews. Molecular cell biology.

[38] Kroemer G (2009). Classification of cell death: recommendations of the Nomenclature Committee on Cell Death 2009. Cell Death Differ.

[39] Schneider CA, Rasband WS, Eliceiri KW (2012). NIH Image to ImageJ: 25 years of image analysis. Nat Meth.

[40] Hevner RF, Daza RA, Englund C, Kohtz J, Fink A (2004). Postnatal shifts of interneuron position in the neocortex of normal and reeler mice: evidence for inward radial migration. Neuroscience.

