# Supplementary material for: Lamin B1 levels modulate differentiation into neurons during embryonic corticogenesis
Source: Sci Rep. 2017 Jul 7;7:4897. doi: 10.1038/s41598-017-05078-6 (PMC5501862; doi:10.1038/s41598-017-05078-6)
Supplement: Supplementary file 1 — Supplemental Materials [file 41598_2017_5078_MOESM1_ESM.pdf]

Lamin B1 levels modulate differentiation into neurons during embryonic corticogenesis

Sameehan Mahajani[1§] , *Caterina Giacomini*[1†] , *Federica Marinaro*[1], *Davide De Pietri Tonelli*[1], *Andrea Contestabile*[1], *Laura Gasparini*[1‡*]

[1]*Dept. of Neuroscience and Brain Technologies, Istituto Italiano di Tecnologia, Genova, Italy.*

[§] current address: Universitaetsmedizin Goettingen, Waldweg 33, Goettingen 37073, Germany

[†]current address: Division of Cancer Studies, King's College London, New Hunt's House, Guy's Campus, London SE1 1UL, UK

[‡]current address: Abbvie Deutschland GmbH & Co, Knollstr, Ludwigshafen 67061, Germany

Running Title: Lamin B1 modulates neuronal differentiation

[*]Corresponding Author
Laura Gasparini
Abbvie Deutschland GmbH & Co
Knoll str.
Ludwigshafen 67061
Germany
e-mail: laura.gasparini@abbvie.com

Supplementary Figure S1-S5

Supplementary Table S1

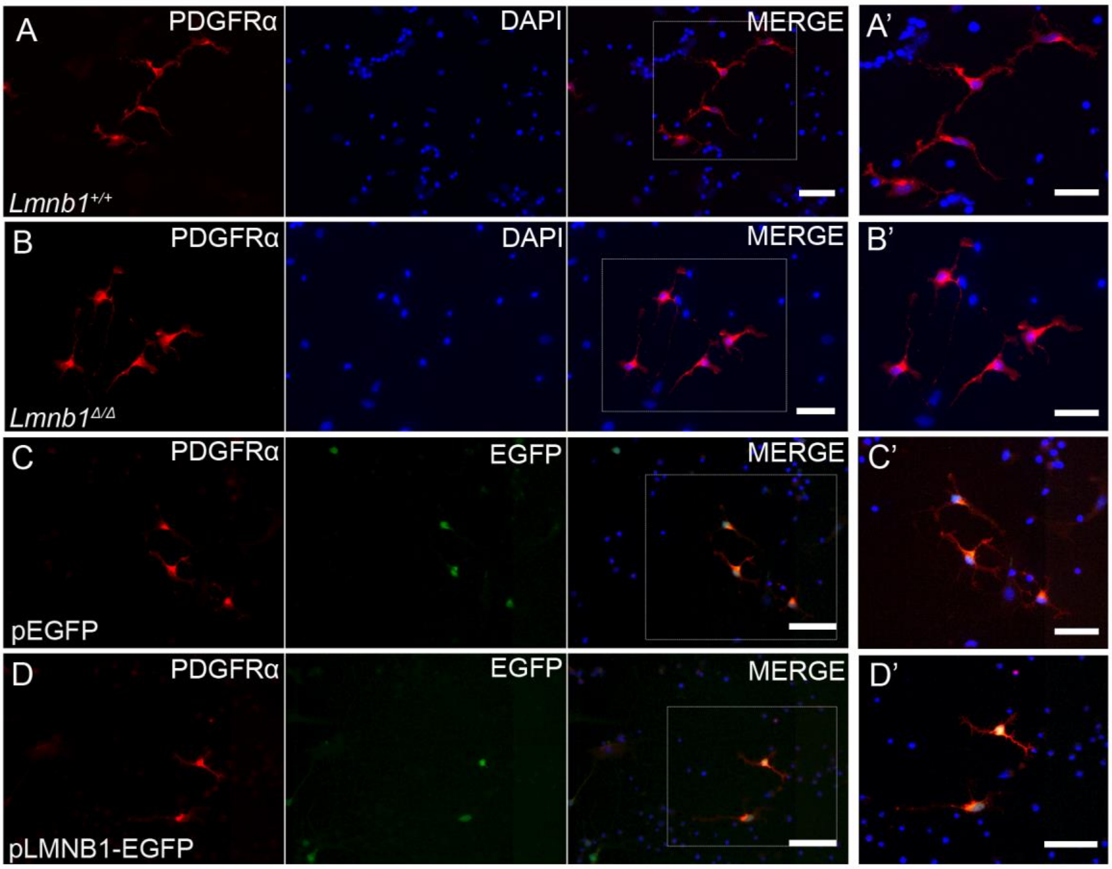

Supplementary Figure S1. Altered levels of Lamin B1 do not affect oligodendrocyte differentiation.

NSCs from *Lmnb1*$^{+/+}$ or *Lmnb1*$^{\Delta/\Delta}$ embryos and WT NSCs transfected with pEGFP or pLMNB1-EGFP plasmids were cultured and differentiated for 4 days. **A-D and A'-D'.** Fluorescence images of PDGFRα immunoreactivity (red) and EGFP (C-D, C'-D', green) in differentiated *Lmnb1*$^{+/+}$ (A, A') or *Lmnb1*$^{\Delta/\Delta}$ (B, B') NSCs and WT NSCs transfected with pEGFP (C, C') or pLMNB1-EGFP (D, D'). In all images, nuclei were counterstained with DAPI (blue). Panels A', B', C' and D' display images at higher magnification of boxed region of A-D. Scale bars: 50 µm.

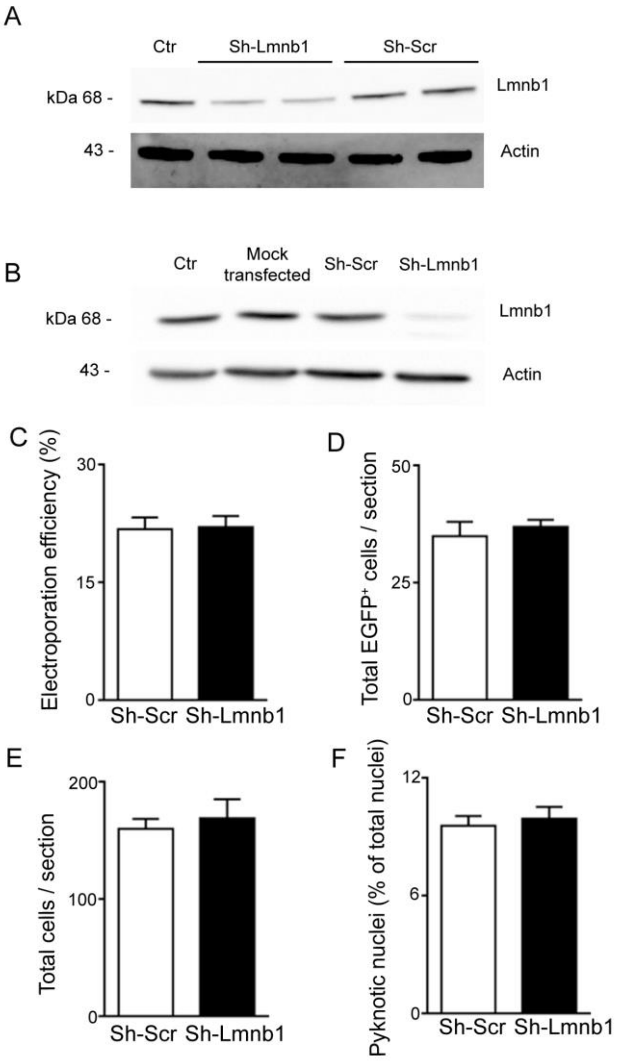

Supplementary Figure S2. In utero electroporation efficiency and apoptotic cell count after Lmnb1 silencing by sh-Lmnb1.

A-B. *Lmnb1* knockdown in N2a cells (A) and primary cortical neurons (B). Cells were transfected or transuced with Sh-Lmnb1, Sh-Scr or mock as described in the methods and analyzed 72 h after transfection or 7 days after transuction. Representative western blot of Lamin B1 and actin are shown. **C.** Electroporation efficiency. Bars represent the percentage of EGFP+ cells over total cells. **D.** Number of EGFP+ per section. **E.** Total number of cells per section. Cells were identified by Hoechst nuclear staining as defined in the methods. **F.** Pyknotic nuclei count. Bars represent the percentage of cells with pyknotic nuclei over total cells per section.

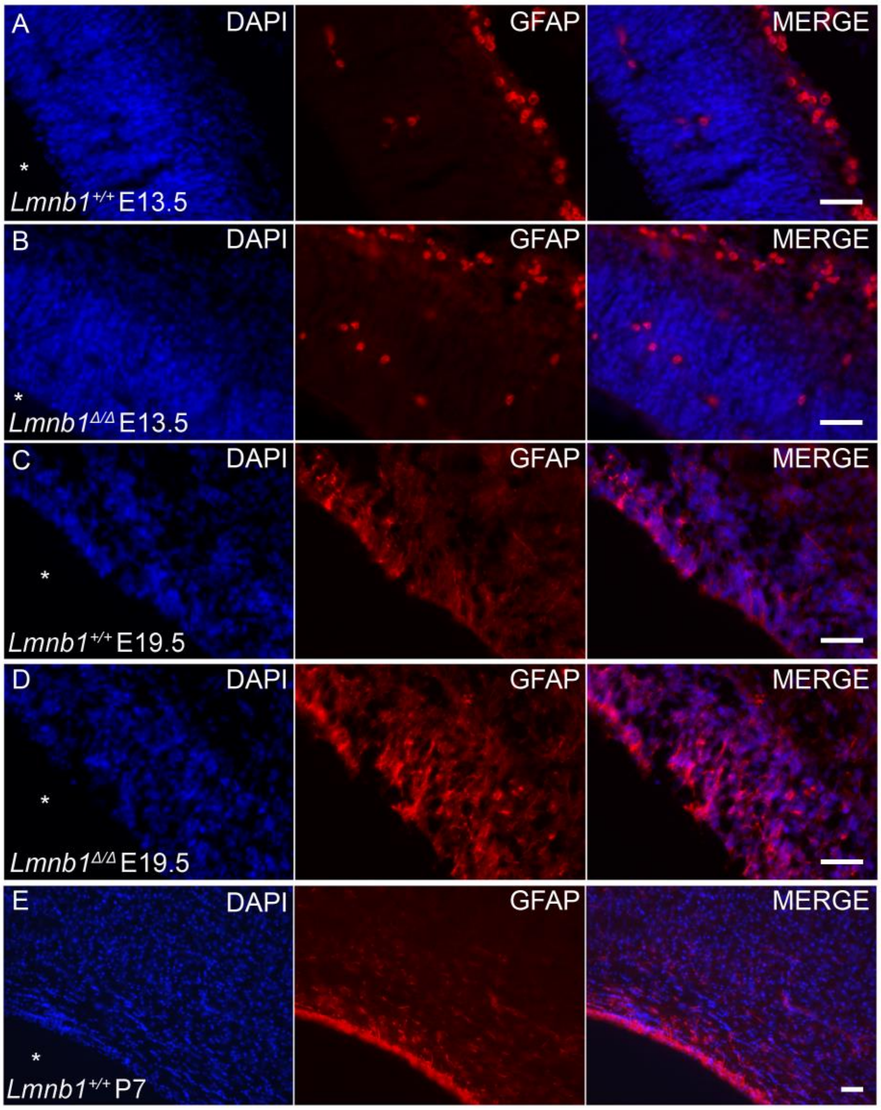

Supplementary Figure S3. GFAP immunoreactivity during brain development in and *Lmnb1*$^{+/+}$ mice.

A-E. Fluorescence images of brain GFAP immunoreactivity (red) in *Lmnb1*$^{+/+}$ E13.5 (A, C, E) and *Lmnb1*$^{\Delta/\Delta}$ (B, D), *Lmnb1*$^{+/+}$ E13.5 (A-B) and E19.5 (C-D) embryos and P7 pups (E). White asterisks identify the ventricle. In all images, nuclei were counterstained with DAPI (blue). Scale bars: 50 µm.

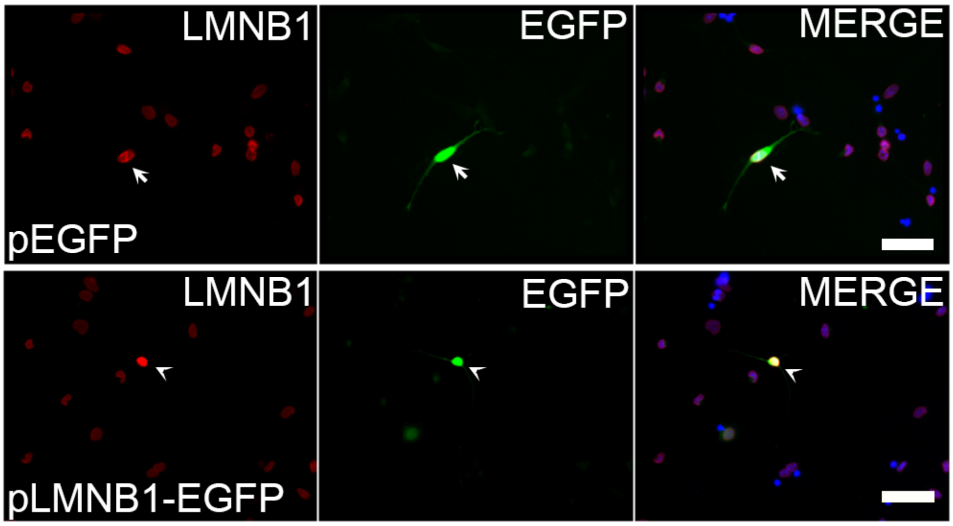

Supplementary Figure S4. LMNB1 overexpression in NSCs.

WT-derived NSCs were transfected with pLMNB1-EGFP or pEGFP plasmids and cultured as described in the Methods. Fluorescence images of immunoreactivity for LMNB1 (red) and EGFP (green) are shown. Nuclei are counterstained with DAPI (blue). Scale bars: 50 µm.

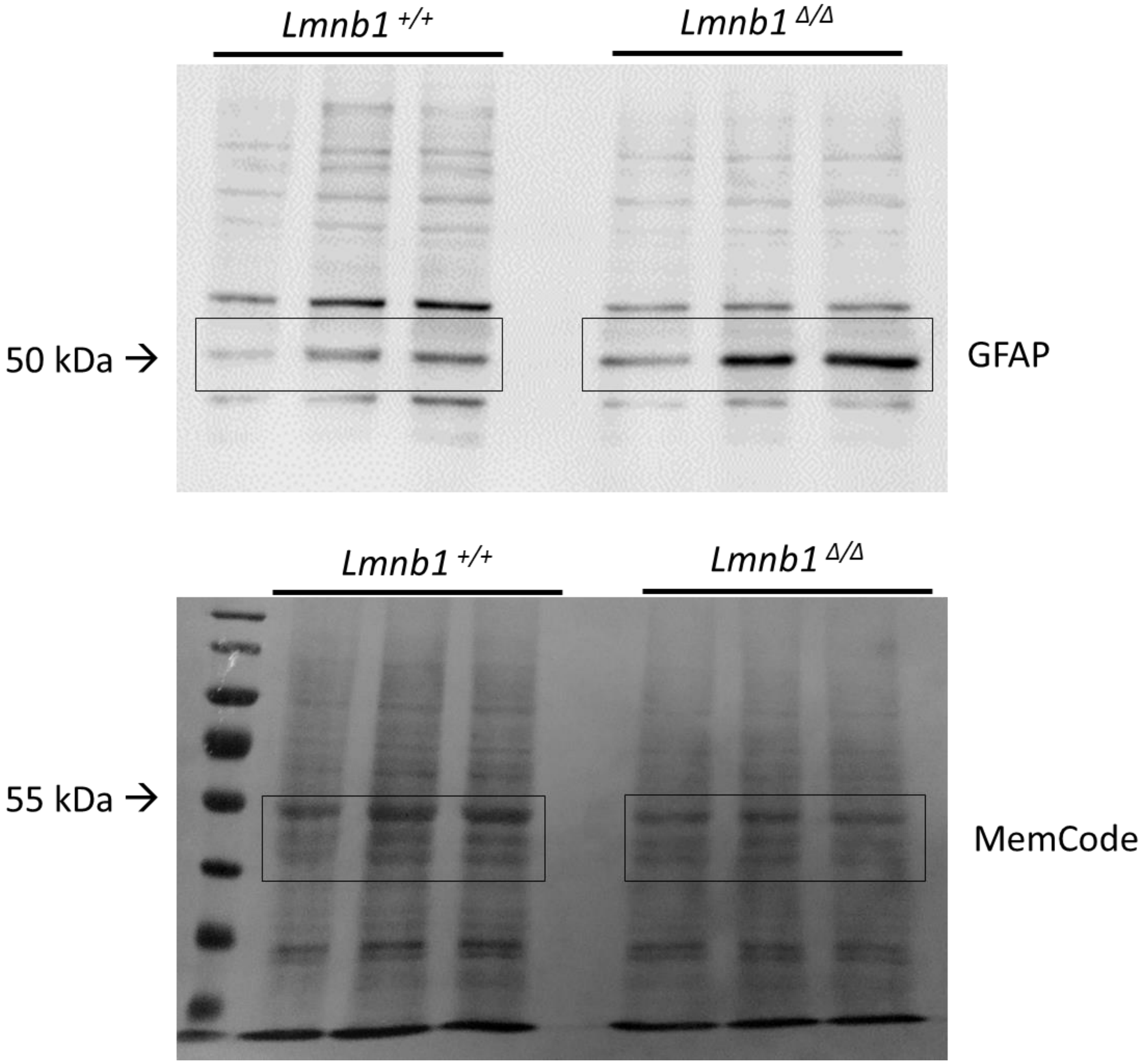

Lmnb1 +/+
Lmnb1 Δ/Δ
50 kDa →
GFAP
Lmnb1 +/+
Lmnb1 Δ/Δ
55 kDa →
MemCode

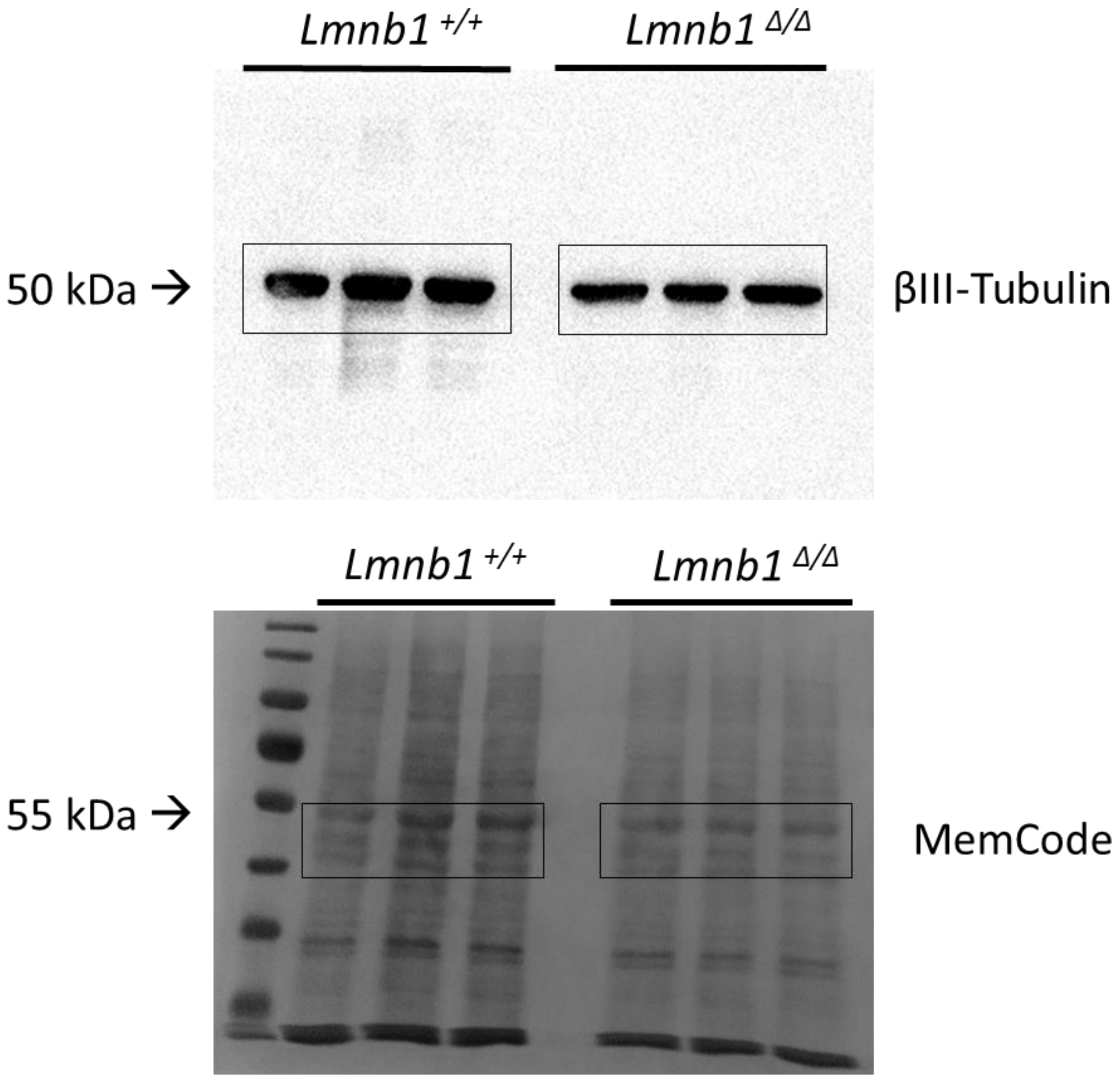

Supplementary Figure S5. Uncropped Western blots as shown in Fig. 4A.

Lmnb1$^{+/+}$ and *Lmnb1*$^{\Delta/\Delta}$ whole brain lysates at E17.5 were checked for GFAP and βIII-Tubulin. Data was normalized to MemCode.

Table S1

Primers used for qRT-PCR analysis

Gene	*Forward primer*	*Reverse primer*	*Efficiency*
Actin	*AAGTGGTTACAGGAAGTCC*	*ATAATTTACACAGAAGCAATGC*	*2.09*
HPRT1	*TGAGGCGGCGAGGGAGAG*	*AAGCGGTCTGAGGAGGAAGC*	*2.08*
GFAP	*GATCTATGAGGAGGAAGTTC*	*CGTATTGAGTGCGAATCT*	*2.05*
βIII-Tubulin	*GCCTTTGGACACCTATTCAGG*	*TTCTTTCCTCAGGACATCCAG*	*2.07*
DCX	*GCCCTTCCTCCTCACTTTCACA*	*GTCTCAATGCCTCCCTCTCCTT*	*2.01*
